# A Polymorphism in the *HLA-DPB1* Gene Is Associated with Susceptibility to Multiple Sclerosis

**DOI:** 10.1371/journal.pone.0013454

**Published:** 2010-10-26

**Authors:** Judith Field, Sharon R. Browning, Laura J. Johnson, Patrick Danoy, Michael D. Varney, Brian D. Tait, Kaushal S. Gandhi, Jac C. Charlesworth, Robert N. Heard, The Australia and New Zealand Multiple Sclerosis Genetics Consortium (ANZgene), Graeme J. Stewart, Trevor J. Kilpatrick, Simon J. Foote, Melanie Bahlo, Helmut Butzkueven, James Wiley, David R. Booth, Bruce V. Taylor, Matthew A. Brown, Justin P. Rubio, Jim Stankovich

**Affiliations:** 1 Florey Neuroscience Institutes, University of Melbourne, Melbourne, Victoria, Australia; 2 Centre for Neuroscience, University of Melbourne, Melbourne, Victoria, Australia; 3 Department of Statistics, The University of Auckland, Auckland, New Zealand; 4 The University of Queensland Diamantina Institute, Princess Alexandra Hospital, University of Queensland, Brisbane, Queensland, Australia; 5 The Australian Red Cross Blood Service, Melbourne, Victoria, Australia; 6 The Westmead Millenium Institute, University of Sydney, Sydney, New South Wales, Australia; 7 Department of Genetics, Southwest Foundation for Biomedical Research, San Antonio, Texas, United States of America; 8 Royal Melbourne Hospital, Melbourne, Victoria, Australia; 9 Menzies Research Institute, University of Tasmania, Hobart, Tasmania, Australia; 10 The Walter and Eliza Hall Institute of Medical Research, Melbourne, Victoria, Australia; 11 Department of Medicine, University of Melbourne, Melbourne, Victoria, Australia; 12 Department of Neurology, Box Hill Hospital, Box Hill, Victoria, Australia; 13 Nuffield Department of Orthopaedic Surgery, Botnar Research Centre, University of Oxford, Oxford, United Kingdom; 14 GlaxoSmithKline, Harlow, Essex, United Kingdom; Hospital Vall d'Hebron, Spain

## Abstract

We conducted an association study across the human leukocyte antigen (HLA) complex to identify loci associated with multiple sclerosis (MS). Comparing 1927 SNPs in 1618 MS cases and 3413 controls of European ancestry, we identified seven SNPs that were independently associated with MS conditional on the others (each 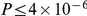). All associations were significant in an independent replication cohort of 2212 cases and 2251 controls (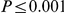) and were highly significant in the combined dataset (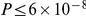). The associated SNPs included proxies for HLA-DRB1*15:01 and HLA-DRB1*03:01, and SNPs in moderate linkage disequilibrium (LD) with HLA-A*02:01, HLA-DRB1*04:01 and HLA-DRB1*13:03. We also found a strong association with rs9277535 in the class II gene *HLA-DPB1* (discovery set 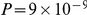, replication set 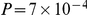, combined 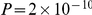). *HLA-DPB1* is located centromeric of the more commonly typed class II genes *HLA-DRB1*, *-DQA1* and *-DQB1*. It is separated from these genes by a recombination hotspot, and the association is not affected by conditioning on genotypes at *DRB1*, *DQA1* and *DQB1*. Hence rs9277535 represents an independent MS-susceptibility locus of genome-wide significance. It is correlated with the HLA-DPB1*03:01 allele, which has been implicated previously in MS in smaller studies. Further genotyping in large datasets is required to confirm and resolve this association.

## Introduction

Multiple sclerosis (MS, OMIM 126200) is an autoimmune disease of the central nervous system which is most prevalent in young adults of European ancestry. It is hypothesized that immune dysregulation leads to autoimmune attack on central nervous system myelin, and that both demyelination and axonal injury play key roles in disability progession. MS aggregates to some extent in families, and is clearly triggered by a complex mix of genetic and environmental factors. In Europeans, the HLA class II haplotype DRB1*15:01–DQB1*06:02 (DR15) is by far the strongest genetic risk factor (odds ratio approximately 3). There is evidence of interaction between this haplotype and environmental risk factors such as vitamin D [1] and exposure to infant siblings [2]. Recently genome-wide association (GWA) studies and follow-up studies have identified many other non-HLA loci associated with MS susceptibility [3], [4], [5], [6], [7], [8], [9], [10], [11], [12], [13], [14]. Most of these loci have been associated with other autoimmune diseases or encompass genes with known immune functions; the notable exceptions are associations with two genes involved in axonal transport [9], [13].

There is now also clear evidence of multiple, independent associations with MS across the major histocompatibility complex (MHC), as is the case for other autoimmune diseases [15], [16]. There have been associations with other alleles of *HLA-DRB1* [17], [18], [19], [20], [21], independent associations in the class I region [4], [16], [22], [23], [24], [25], [26], [27], and cis [28] and trans [17], [29] interactions between alleles of class II genes. The outstanding candidate genes across the MHC are the highly polymorphic class II and class I genes which present antigens to CD4+ and CD8+ T cells respectively, but there are many other compelling candidates in the HLA complex, which is rich in genes of immune function. Elucidating causative genes and polymorphisms is harder than elsewhere in the genome due to the extensive linkage disequilibrium (LD) across the region.

To find evidence of independent associations, it is necessary either to perform stratified analyses or to condition on other associations. In a recent study, dense SNP genotyping data across the MHC was explored by conditioning on the predominant DRB1*15:01 association [16]. In the current study we extend this approach, conditioning on multiple MS-associated SNPs and imputed MHC alleles (in addition to DRB1*15:01) to identify further independent associations and build up multivariate models of MS-susceptibility. SNPs and alleles in these models were then validated by genotyping them (or proxies for them) in an independent replication case-control dataset.

## Results

### A multivariate SNP model of MS-susceptibility

**Figure 1:**
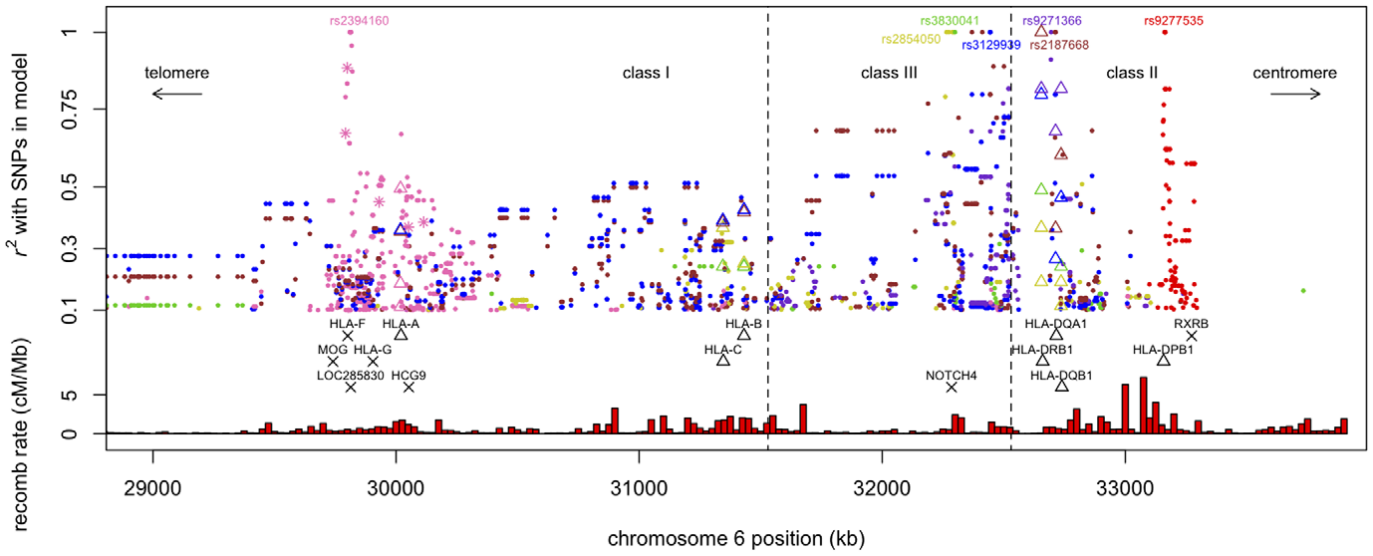
LD between the seven SNPs in the fitted model (**Table 1**) and other MHC polymorphisms. The plot shows LD between the 7 model SNPs and other SNPs and classical HLA alleles genotyped in the HapMap CEU population. Points are only plotted for SNP pairs where 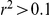. Points are plotted at the genomic positions of the HLA alleles/other SNPs; named points at the top of the plot (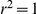) show the locations of the SNPs in the model. The various colours are used to distinguish LD with different SNPs in the model (rs2394160 pink, rs2854050 khaki, rs3830041 green, rs3129939 blue, rs9271366 purple, rs2187668 brown, rs9277535 red). SNPs are shown as small dots and HLA alleles are shown as triangles: refer to Table 2 for the names of HLA alleles correlated with 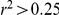. Pink stars denote SNPs in LD with rs2394160 that were strongly associated with MS in the second stage of the model-building (just with adjustment for the DRB1*15:01 proxy SNP rs9271366). The black triangles and crossess show the locations of some classical HLA genes and other genes respectively that are mentioned in the text. The red bars at the bottom of the graph show recombination rates averaged over 25kb windows. The vertical dashed lines mark the boundaries of the class I, class III and class II regions of the MHC.

Our discovery dataset consisted of the genotypes of 1618 MS cases and 3413 controls of European ancestry at 1927 SNPs across the MHC and flanking regions, samples which were previously analysed in a genome-wide association study [6]. We began by testing all SNPs individually for association with MS using a trend test. The most strongly associated SNP was rs9271366, a proxy for the HLA-DRB1*15:01 allele (genotypic squared correlation 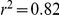 in HapMap CEU data [30], 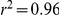 with DR15 in two-digit typing of 740 cases from the discovery dataset). In the second step, we tested all other SNPs using a trend test, conditioning on genotype at rs9271366 as a three-level factor. The most significantly associated SNP was rs2394160 (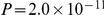), located in an intron of *LOC285830* approximately 15 kb centromeric of the *HLA-F* gene in the class I region. This SNP is in strong LD (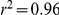) with MS-associated SNPs rs2743951 and rs2523393 reported in previous studies [4], [16]. There were six other SNPs with P-values less than 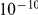: five SNPs correlated with rs2394160 (rs2517912, rs1262126, rs2975033, rs6904029, rs4711207, see Figure 1) and rs2854050 in an intron of *NOTCH4*.

In the third step, all SNPs were tested conditioning on genotypes at rs9271366 and rs2394160. The strongest association was with rs2854050. In subsequent steps we identified four more independently-associated SNPs (rs3830041, rs3129939, rs2187668, rs9277535), including a proxy (rs2187668) for the HLA-DRB1*03:01 allele (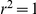 in HapMap CEU) which has been associated with MS previously [17], [18], [19], [20], [29]. No further SNPs were significant after applying a Bonferroni correction for 1927 tests. Table 1 shows a fitted model containing additive terms for these seven SNPs.

**Table 1:** Seven SNPs across the MHC independently associated with MS: terms in the fitted logistic regression model.

				Discovery dataset: 1618 cases, 3413 controls				Replication dataset: 2212 cases, 2251 controls				Combined^3^			
SNP	Position^1^	Minor allele	Major allele	MAF cases	MAF controls	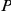 value	OR	MAF cases	MAF controls	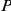 value^2^	OR	MAF cases	MAF controls	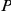 value	OR
rs2394160	29,811,241	G	A	0.344	0.423	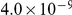	0.75	0.359	0.437	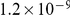	0.75	0.353	0.429	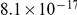	0.76
rs2854050	32,293,583	A	G	0.035	0.072	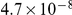	0.53	0.039	0.070	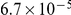	0.67	0.037	0.071	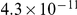	0.60
rs3830041	32,299,317	A	G	0.083	0.086	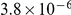	1.50	0.074	0.084	0.0014	1.31	0.077	0.085	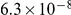	1.41
rs3129939	32,444,744	G	A	0.159	0.189	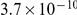	0.56	0.166	0.186	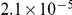	0.69	0.163	0.188	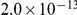	0.62
rs9271366	32,694,832	G	A	0.329	0.155	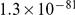	3.04	0.339	0.151	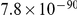	3.38	0.334	0.153	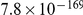	3.19
rs2187668	32,713,862	A	G	0.146	0.136	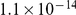	2.15	0.148	0.135	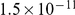	1.88	0.147	0.136	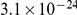	2.01
rs9277535	33,162,839	G	A	0.273	0.240	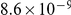	1.36	0.265	0.261	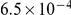	1.18	0.269	0.249	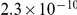	1.27

1 SNP positions are from NCBI dbSNP genome build 128 (October 2007).

2 One sided 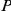 values are used for the replication analysis only.

3 Analysis of the combined dataset was adjusted for sample group (discovery/replication).

One might expect the form of this model to be heavily influenced by the terms included in the model-building procedure (the 1927 SNPs in the discovery dataset). However when we repeated the model-building procedure, augmenting the 1927 SNPs with 10,260 haplotype clusters selected using a variable-length Hidden Markov Model of LD [31], [32], the fitted model was remarkably similar (data not shown). This SNP-plus-haplotype model included 6 terms: 4 SNPs from the SNP-only model (rs2394160, rs2854050, rs9271366, rs9277535), and two 2-SNP haplotypes which each included a SNP from the SNP-only model (rs2854050, rs2187668). Hence we focused on the SNP-only model for replication and interpretation.

We attempted to replicate the 7-SNP model (Table 1) by genotyping these SNPs, and others across the MHC, in an independent dataset comprising 2212 cases and 2251 controls. In the replication data all terms were significant after correcting for multiple testing (one-sided 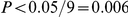, Table 1); hence they were all highly significant in the combined dataset (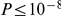). In the combined dataset we tested for departures from multiplicativity (dominance effects) and interactions with the DRB1*15:01 proxy rs9271366. There were significant dominance effects for the DRB1*15:01 and DRB1*03:01 proxy SNPs, and there was modest evidence of interaction (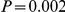) between rs2854050 and rs9271366 (Table S1).

### Correlations with classical MHC alleles

The seven SNPs in the SNP-only model (Table 1) are in LD with many other polymorphisms across the MHC. Figure 1 shows LD (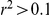) between the SNPs in the model and other SNPs, as well as alleles of the classical MHC genes *HLA-A*, *HLA-B*, *HLA-C*, *HLA-DRB1*, *HLA-DQA1* and *HLA-DQB1*, in 90 unrelated individuals from the HapMap CEU population [30]. Table 2 shows classical alleles correlated with 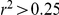.

**Table 2:** Alleles of the classical MHC genes *HLA-A*, *HLA-C*, *HLA-B*, *HLA-DRB1*, *HLA-DQA1* and *HLA-DQB1* correlated with SNPs in the fitted logistic regression model (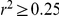).

SNP in model	correlated classical allele	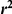 in HapMap CEU
rs2394160	HLA-A*02:01	0.49
rs2854050	HLA-C*05:01	0.37
	DRB1*04:01	0.37
	HLA-B*44:02	0.25
rs3830041	DRB1*13:03	0.49
rs3129939	DRB1*03:01	0.80
	DQB1*02:01	0.47
	HLA-B*08:01	0.43
	HLA-C*07:01	0.39
	HLA-A*01:01	0.36
	DQA1*05:01	0.27
rs9271366	DRB1*15:01	0.82
	DQB1*06:02	0.82
	DQA1*01:01	0.68
rs2187668	DRB1*03:01	1.00
	DQB1*02:01	0.60
	HLA-B*08:01	0.42
	HLA-C*07:01	0.38
	DQA1*05:01	0.37
	HLA-A*01:01	0.36
rs9277535	none	

We investigated the effects of some of these HLA alleles, by imputing them in the discovery dataset and genotyping proxy SNPs in the replication dataset. We added imputed allele dosages and proxy SNPs to the logistic regression model one-by-one, both with and without the corresponding correlated SNP in the original model (see Table S2 for imputed dosages and Table S3 for proxy SNPs).

These analyses provided evidence that associations with the three most telomeric model SNPs (rs2394160, rs2854050 and rs3830041) may be driven by LD with MHC alleles that have been associated with MS previously: HLA-A*02:01 (protective) [22], [23], [33], DRB1*04:01 (protective) [20], [34] and DRB1*13:03 (susceptibility) [21], [35] respectively. In the discovery dataset, imputed dosages for these three HLA alleles show strong associations when they are substituted for the relevant SNP (HLA-A*02:01, 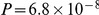; DRB1*04:01, 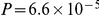; DRB1*13:03, 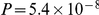, Table S2). In the discovery dataset rs6904029, which was strongly associated in the second step of the model building (pink stars in Figure 1), is strongly correlated with HLA-A*02:01 (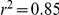). In the replication dataset the association with rs2394160 completely disappears (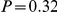) after adjustment for a good proxy for HLA-A*02:01 (rs2523822, 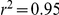, Table S3). This SNP is highly significant in the model, both with and without adjustment for rs2394160 (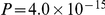 and 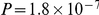 respectively). After adjustment for rs2394160 in the replication dataset, there is little evidence of association with candidate polymorphisms rs2857766 and rs1233334 in the nearby genes *MOG* [22], [36] and *HLA-G* [37] (Table S3).

These analyses (Table S2) did not provide any evidence that the association with rs3129939 is caused by LD with correlated alleles of *HLA-A*, *HLA-B*, *HLA-C*, *HLA-DRB1*, *HLA-DQA1* or *HLA-DQB1* (Table 2), and rs9277535 shows low correlation with all alleles of these genes. Table 3 shows an alternative logistic regression model fitted to the discovery dataset, with the other five SNPs replaced by imputed dosages for correlated HLA alleles.

**Table 3:** Alternative logistic regression model fitted to the discovery dataset, with five SNPs from the model in Table 1 replaced by imputed dosages for correlated MHC alleles.

Term in model	Replaced SNP	Freq cases	Freq controls	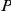 value	OR
HLA-A*02:01	rs2394160	0.200	0.266	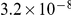	0.72
DRB1*03:01	rs2187668	0.153	0.143	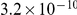	1.84
DRB1*04:01	rs2854050	0.055	0.102	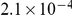	0.66
DRB1*13:03	rs3830041	0.021	0.011	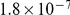	2.65
DRB1*15:01	rs9271366	0.324	0.153	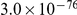	3.05
rs3129939G	–	0.159	0.189	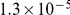	0.67
rs9277535G	–	0.273	0.240	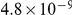	1.36

### Association with a SNP in the gene *HLA-DPB1*

rs9277535 lies in the 3¿ untranslated region of another class II gene, *HLA-DPB1*. All moderately correlated SNPs (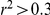, Figure 1) lie within a 131-kb region (33,155kb–33,286kb) which also encompasses the genes *HLA-DPB2*, *COL11A2*, *RXRB*, *SLC39A7*, *HSD17B8*, *MIR219-1* and *RING1*. *HLA-DPB1* is centromeric of the class II genes *HLA-DRB1*, *HLA-DQA1* and *HLA-DQB1*, and is separated by a region with a high recombination rate (Figure 1). Consequently, LD is low between rs9277535 and all *DRB1*, *DQA1* and *DQB1* alleles in the HapMap CEU dataset (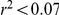). In the discovery dataset, the association with rs9277535 remains strong after conditioning on imputed allele dosages at all alleles of *DRB1*, *DQA1* and *DQB1* (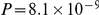), and with full conditioning on maximum likelihood genotypes at *DRB1* (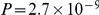); hence the association is unlikely to be caused by complex interactions between *DRB1* alleles [17], [29]. There was no evidence that rs9277535G exerts different effects in DR15-positive and -negative individuals (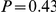 testing for interaction with rs9271366 in the combined dataset).

The association with rs9277535 is somewhat masked due to negative confounding by DRB1*15:01, even though LD between the *DRB1* and *DPB1* loci is weak. Table S4 shows correlations between the risk-associated rs9277535 allele and imputed allele dosages for various classical alleles in the discovery dataset. In particular, rs9277535G is negatively-correlated with the DRB1*15:01 allele (*r* = ¿0.09). In an unadjusted analysis of this SNP in the discovery dataset, the estimated odds ratio for rs9277535G is 1.19 (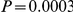). The estimated odds ratio increases to 1.34 with adjustment for DRB1*15:01 and to 1.36 in the full model (Table 3) after further adjustment for DRB1*03:01 (negative confounder) and DRB1*04:01 (positive confounder). In the replication dataset, negative confounding means that the association with rs9277535 is significant after adjustment for other terms, even though the overall frequency of the minor allele is very similar in cases and controls (0.265 and 0.261 respectively).

A recent study showed that there is a highly-conserved vitamin D response element (VDRE) GGGTGGAGGGGGTTCA immediately upstream of the *HLA-DRB1* gene on DRB1*15:01 haplotypes [1]. We searched for Vitamin D Response Elements (VDREs) associated with the HLA-DPB1*03:01 allele [38], using the homozygous COX cell line which carries this allele. This in silico analysis revealed one predicted VDRE. However this sequence differed from the sequence proximal to HLA-DRB1*15:01, was not located in the proximal promoter region of HLA-DPB1*03:01 and was found in all *HLA-DPB1* alleles assessed.

### Association with a copy number variant

We observed a copy number variant in the course of genotyping the HLA-B*44:02 proxy SNP rs2256583C
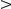
T in the replication dataset. This SNP lies between *HLA-C* and *HLA-B* in a region known to contain copy number variants. Plotting areas under the C- and T-allele peaks revealed three groups of heterozygotes, which appear to correspond to C∶T ratios of 2∶1, 1∶1 and 1∶2 (Figure S1). There were different proportions of cases in the three groups (Table S5, 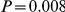 using Fisher's exact test). The heterozygote groupings are not well-correlated with any of the seven SNPs in the model of Table 1, and these differences remain almost significant after adjustment for all terms in the SNP model (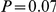 using a likelihood ratio test with two degrees of freedom). There was too much inter-sample variation in peak heights to detect copy number differences among homozygotes.

## Discussion

Using multivariate analysis we identified seven SNPs that were independently associated with MS, and confirmed all seven associations in a replication dataset. All associations were highly significant in the combined dataset (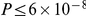, Table 1).

Two of these SNPs are proxies for the known associations with DRB1*15:01 and DRB1*03:01. As in previous analyses [4], [16], the most prominent associations after conditioning on DRB1*15:01 were with SNPs in the vicinity of *HLA-A*. Hence our data add to previous, very strong evidence of a functional effect correlated with rs2394160, rs2743951 [16], rs2523393 [4], rs6904029 and HLA-A*02:01 [33]. We also found evidence to support previously-reported associations with the alleles DRB1*04:01 [20], [34] and DRB1*13:03 [21], [35].

In MS, strong protective associations have been reported previously for the class I alleles HLA-C*05:01 [26] and HLA-B*44:02 [16], which are highly correlated with one another (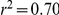 in HapMap CEU data). In an unadjusted analysis, HLA-B*44:02 is strongly associated with MS in our discovery dataset (OR 0.67, 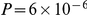), however this association is positively confounded by the DRB1*15:01 association (*r* = ¿0.05), and by the (putative) protective effects of HLA-A*02:01 (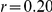) and DRB1*04:01 (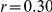). Hence, HLA-B*44:02 shows little evidence of association when added to the model in Table 3 (OR = 0.92, 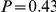). Our data provide stronger evidence of an association with DRB1*04:01 than with HLA-C*05:01 or HLA-B*44:02, but this evidence is far from definitive. The multivariate analyses suggest that not all three of these alleles are exerting causative effects, but further analyses in larger datasets and functional studies will be required to determine which (if any) are causative.

The most novel association in the model is with the SNP rs9277535 in the class II gene *HLA-DPB1*, centromeric of the DR/DQ region and separated by a recombination hotspot. The DP region has not been investigated as thoroughly as the DR/DQ region in genetic association studies. In normal conditions DP gene products are expressed at much lower levels than DR gene products [39]; therefore DP typing was difficult with serological techniques prior to the advent of molecular typing. Associations with DR and DQ alleles were detected early on for many diseases including MS [40], so there has been a natural focus on these genes.

Nevertheless there have been several studies reporting associations between MS and *HLA-DPB1*. A SNP rs3135021 in an intron of *HLA-DPB1* was recently associated with MS in African Americans [41]. The SNP is in LD with some *HLA-DRB1* alleles in African Americans [41], and it is in linkage equilibrium (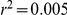) with rs9275535 in the HapMap CEU population. The DPB1*05:01 allele has been associated with opticospinal MS in Japan [42] and with MS in Southern Han Chinese [43]. The DPB1*03:01 allele has been implicated in epitope spreading in MS [44]. It has also been associated with opticospinal MS in Japan [45], and with MS in a small Australian study [46] and in Sardinia [35]. In the Sardinian study, comparing 835 relapsing MS patients with 592 controls, carriage of the DPB1*03:01 allele was associated with increased risk of MS (OR 1.30, 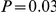) after conditioning on an associated microsatellite in the class I region and on presence or absence of five *DRB1–DQB1* risk haplotypes (three of which include DRB1*15:01, DRB1*03:01 and DRB1*13:03). We tested for presence/absence of the DPB1*03:01 allele in 422 Australian MS cases using a combination of sequence specific priming and sequence based typing, and found a correlation with rs9277535 (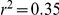, Table S6): all but two individuals carrying the DPB1*03:01 allele also carry the rs9277535G allele.

Mechanisms other than peptide affinities for HLA molecules may be underpinning some of these associations. The HLA complex has a particularly high density of copy number variants, and these represent one possible source of functional variation. In the course of replication genotyping we identified a copy number variant between the genes *HLA-B* and *HLA-C* which showed some evidence of independent association with MS susceptibility.

Disease-associated variants may also be acting via effects on gene expression [47]. A recent study suggests that a vitamin D response element (VDRE) upstream of *HLA-DRB1* plays an important role in expression of this gene [1]. VDREs are bound by the vitamin D receptor (VDR) which acts as a ligand activated transcription factor, and the VDRE upstream of *HLA-DRB1* is highly conserved on DRB1*15:01 haplotypes. It was found that the DRB1*15:01 haplotype binds the VDR more efficiently than other haplotypes, in particular the 98% of DRB1*04, DRB1*07 and DRB1*09 haplotypes where this sequence differs at two nucleotides (GGGTGGAG**A**GGG**G**TCA) [1]. We observed that the homozygous COX and QBL lymphoblastoid cell lines, which both carry the risk-associated HLA-DRB1*03:01 allele, have an identical proximal VDRE to that occurring on the DRB1*15:01 haplotype. This is noteworthy given that the amino acid sequences of DRB1*15:01 and DRB1*03:01 are not particularly similar compared to other *DRB1* alleles.

We also searched for VDREs associated with the HLA-DPB1*0301 allele, but did not find convincing evidence of an active response element. One of the genes in the region of LD around *HLA-DPB1* is retinoid X receptor beta (*RXRB*). This gene encodes a receptor which forms a dimer with VDR and increases its DNA binding and transcriptional function [48].

In summary, we have detected a highly significant association with MS in the vicinity of the *HLA-DPB1* gene that is independent of other MS associations in the HLA complex, confirming suggestive reports from previous smaller studies. Our data indicate that DPB1*03:01 may be the causative HLA allele underpinning this association, however positive replication studies followed by fine mapping will be required to confirm and resolve this association in other populations. Our study also highlights the utility of multivariate analysis to disentangle HLA associations for autoimmune diseases, such as MS, which have multiple genetic risk factors in the HLA complex. This approach will be useful more generally at other loci and for other diseases, as more clusters of associations are identified [14], [49].

## Materials and Methods

Approval for this research was granted by the Melbourne Health Human Research Ethics Committee and other institutional ethics committees. Written consent was given by the patients for their information to be stored in the study database and used for research.

To identify SNPs across the MHC independently associated with MS, we analysed genotype data assembled for a GWA study [6]. After data cleaning followed by principal components analysis to remove population outliers [6], this dataset comprised 1618 MS cases of European ancestry from Australia and New Zealand, and 3413 European-ancestry controls from Australia, the UK and US. All samples were genotyped with Illumina arrays.

The cleaned discovery dataset included genotypes at 1927 SNPs across the MHC and flanking regions (chr6: 24–36 Mb, NCBI dbSNP genome build 128). We used a stepwise logistic regression procedure to identify SNPs independently associated with disease status, building a model with increasing numbers of independently associated SNPs. At each step we used a trend test to identify the most significantly associated SNP after conditioning on SNPs already in the model, then added this SNP to the model. Conditioning on SNPs already in the model was performed by coding their genotypes as three-level factors. The procedure was repeated until no further SNPs were significant after applying a Bonferroni correction to correct for 1927 tests. In the final model fitted to the discovery dataset (Table 1), all SNPs in the model were coded as quantitative variables (trend test) rather than factors. The final model was also fitted to the discovery dataset with adjustment for position along the first three eigenvectors of the final principal components analysis [6]; with this additional adjustment no coefficients of SNPs changed by more than 7% and all remained significant at 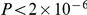 (full data not shown).

Twenty-five SNPs across the HLA region were genotyped in an independent replication dataset of MS cases and controls from Australia and New Zealand described previously [6], using the Sequenom MassARRAY system and iPlex Gold chemistry. These SNPs included the seven SNPs in the fitted model (Table 1), candidate polymorphisms in the genes *MOG* and *HLA-G*, proxies for several classical MHC alleles, and backups in case of assay failure. After excluding 147 samples with call rates less than 90%, the filtered replication dataset comprised 2212 cases and 2251 controls. In this filtered dataset call rates were above 99.7% for all 25 SNPs.

We fitted the same 7-SNP logistic regression model to the replication dataset and to the combined dataset. Two-sided 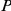 values were used throughout except in the analysis of the replication dataset. Analysis of the combined dataset was adjusted for sample group (discovery/replication). In the replication dataset we also fitted a 7-SNP model using an alternative proxy rs3135388 for HLA-DRB1*15:01 [30]; in this alternative model the magnitudes of the coefficients of rs2854050 and rs3830041 decreased by 32% and 13% respectively but remained significant (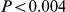), and no other coefficients changed by more than 6% (full data not shown).

To investigate LD with polymorphisms not genotyped in the discovery dataset we downloaded phased data for individuals from the HapMap CEU population (Utah residents with ancestry from northern and western Europe), including classical HLA typing data for the genes *HLA-A*, *HLA-B*, *HLA-C*, *HLA-DRB1*, *HLA-DQA1* and *HLA-DQB1* (http://www.inflammgen.org) [30]. In the discovery dataset we imputed genotypes at classical HLA alleles using the software BEAGLE (http://www.stat.auckland.ac.nz/bbrowning/beagle/beagle.html) [50]. Imputation was done by dividing the samples randomly into batches of 50, and running each batch with the phased HapMap CEU data. The default settings in BEAGLE were changed to allow consideration of long haplotypes spanning the entire region, which has proven beneficial for imputation of HLA alleles [51]. For each classical allele we calculated posterior allele dosage, which is twice the posterior probability of carrying two copies of the allele plus the posterior probability of carrying exactly one copy of the allele. These posterior allele dosages were used as predictive variables in logistic regression models (Table 3, Table S2).

MHC sequences for HLA-homozygous cell lines were downloaded from the website of the MHC Haplotype project (https://www.sanger.ac.uk/HGP/Chr6/MHC/). We searched for VDREs associated with the HLA-DPB1*03:01 allele using the sequence for this allele (carried by the homozygous cell line COX). The sequence was scanned for VDREs using JASPAR (http://jaspar.genereg.net) [38] with a profile threshold of 80%.

## Supporting Information

Table S1 The 7-SNP model in the combined dataset, with two significant dominance effects and one significant SNP-SNP interaction added
(0.02 MB PDF)

Table S2 The effects of adding correlated MHC alleles to the fitted model in the discovery dataset (Table 1), both with and without the correlated SNP. For these analyses imputed MHC allele dosages were calculated using the software BEAGLE (50).
(0.03 MB PDF)

Table S3 The effects of adding other SNPs genotyped in the replication dataset to the fitted model (Table 1), both with and without the correlated SNP from the model.
(0.03 MB PDF)

Table S4 Correlations between various SNPs and imputed classical alleles in the discovery dataset
(0.01 MB PDF)

Table S5 A copy number variant at SNP rs2256583: numbers of heterozygous cases and controls with various C∶T allele ratios in the replication dataset
(0.01 MB PDF)

Table S6 Correlation between HLA-DPB1*0301 and rs9277535 in 422 MS cases
(0.01 MB PDF)

Figure S1 A copy number variant at SNP rs2256583. A plot of area under the C- allele peak (y-axis) versus area under the T-allele peak (x-axis) for heterozygotes, showing three groups of individuals with C∶T ratios of 1∶2 (red), 1∶1 (green) and 2∶1 (blue).
(0.16 MB PDF)
