# Supplementary material for: A Polymorphism in the HLA-DPB1 Gene Is Associated with Susceptibility to Multiple Sclerosis
Source: PLoS One. 2010 Oct 26;5(10):e13454. doi: 10.1371/journal.pone.0013454 (PMC2964313; doi:10.1371/journal.pone.0013454)
Supplement: Table S1 — The 7-SNP model in the combined dataset, with two significant dominance effects and one significant SNP-SNP interaction added (0.02 MB PDF) [file pone.0013454.s001.pdf]

Table S1. The 7-SNP model in the combined dataset, with two significant dominance effects and one significant SNP-SNP interaction added

Term in model	P value	OR
rs2394160G	2.2×10^{-17}	0.75
rs2854050A	6.9×10^{-4}	0.72
rs3830041A	2.2×10^{-8}	1.42
rs3129939G	1.9×10^{-13}	0.62
rs9271366G	4.7×10^{-139}	3.61
rs2187668A	3.5×10^{-15}	1.81
rs9275535G	1.5×10^{-10}	1.27
rs9271366GG	0.0014	0.65[1]
rs2187668AA	3.6×10^{-4}	1.80[1]
rs9271366G × rs2854050A	0.0022	0.66

[1]Odds ratios for dominance terms give the change in odds for minor-allele homozygotes, compared to what would be expected under a multiplicative model. Hence the estimated odds ratio for rs9271366GG homozygotes compared to rs9271366AA homozygotes is $3.61 \times 3.61 \times 0.65 = 8.47$.
