# Supplementary material for: A Polymorphism in the HLA-DPB1 Gene Is Associated with Susceptibility to Multiple Sclerosis
Source: PLoS One. 2010 Oct 26;5(10):e13454. doi: 10.1371/journal.pone.0013454 (PMC2964313; doi:10.1371/journal.pone.0013454)
Supplement: Table S2 — The effects of adding correlated MHC alleles to the fitted model in the discovery dataset (Table 1), both with and without the correlated SNP. For these analyses imputed MHC allele dosages were calculated using the software BEAGLE (50). (0.03 MB PDF) [file pone.0013454.s002.pdf]

Table S2. The effects of adding correlated MHC alleles to the fitted model in the discovery dataset (Table 1), both with and without the correlated SNP. For these analyses imputed MHC allele dosages were calculated using the software BEAGLE (50).

				P-values with both in model		P-values with other not in model	
SNP in model	correlated MHC allele	r^2 in HapMap	Imputation quality[1]	SNP	MHC allele	SNP[2]	MHC allele
rs2394160	HLA-A*0201	0.49	0.95	0.0014	0.025	4.0×10^{-9}	6.8×10^{-8}
rs2854050	HLA-C*0501	0.37	0.89	5.1×10^{-8}	0.47	4.7×10^{-8}	0.21
	DRB1*0401	0.37	0.70	2.1×10^{-5}	0.12	4.7×10^{-8}	6.6×10^{-5}
	HLA-B*4402	0.25	0.88	1.3×10^{-7}	0.99	4.7×10^{-8}	0.084
rs3830041	DRB1*1303	0.49	0.92	0.0095	2.0×10^{-4}	3.8×10^{-6}	5.4×10^{-8}
rs3129939	DQB1*0201	0.47	0.95	2.5×10^{-10}	0.88	3.7×10^{-10}	0.86
	HLA-B*0801	0.43	0.92	8.7×10^{-10}	0.65	3.7×10^{-10}	0.11
	HLA-C*0701	0.39	0.87	1.6×10^{-10}	0.28	3.7×10^{-10}	0.76
	HLA-A*0101	0.36	0.91	1.7×10^{-9}	0.52	3.7×10^{-10}	0.041
	DQA1*0501	0.27	0.94	5.6×10^{-10}	0.48	3.7×10^{-10}	0.13

[1]Imputation was done by combining batches of 50 samples with the HapMap samples; this is an estimate of the average r^2 over the batches between true genotypes and imputed genotypes with the highest posterior probabilities

[2]This is the same model as in Table 1
