# Supplementary material for: A Polymorphism in the HLA-DPB1 Gene Is Associated with Susceptibility to Multiple Sclerosis
Source: PLoS One. 2010 Oct 26;5(10):e13454. doi: 10.1371/journal.pone.0013454 (PMC2964313; doi:10.1371/journal.pone.0013454)
Supplement: Table S3 — The effects of adding other SNPs genotyped in the replication dataset to the fitted model (Table 1), both with and without the correlated SNP from the model. (0.03 MB PDF) [file pone.0013454.s003.pdf]

Table S3. The effects of adding other SNPs genotyped in the replication dataset to the fitted model (Table 1), both with and without the correlated SNP from the model.

SNP in model	Other SNP	Reason for genotyping other SNP	P-values with both in model: model SNP	P-values with both in model: other SNP	P-values with other not in model: model SNP[1]	P-values with other not in model: other SNP
rs2394160	rs2857766	Missense SNP in candidate gene MOG	2.6×10^{-6}	0.95	1.2×10^{-9}	1.8×10^{-4}
	rs1233334	Candidate SNP in promoter of HLA-G	2.4×10^{-8}	0.96	1.2×10^{-9}	0.031
	rs2523822	Proxy for HLA-A*0201 ($r^2 = 0.95$)	0.32	1.8×10^{-7}	1.2×10^{-9}	4.0×10^{-15}
rs2854050	rs2524083	Proxy for HLA-C*0501 ($r^2 = 0.88$)	1.3×10^{-4}	0.52	6.7×10^{-5}	0.50
	rs2256583	Proxy for HLA-B*4402 ($r^2 = 0.91$)	8.4×10^{-5}	0.39	6.7×10^{-5}	0.80
	rs2070600	Rough proxy for DRB1*0401 ($r^2 = 0.48$)	0.0058	0.62	6.7×10^{-5}	0.0061

[1] This is the same model as in Table 1
