# Supplementary material for: A Polymorphism in the HLA-DPB1 Gene Is Associated with Susceptibility to Multiple Sclerosis
Source: PLoS One. 2010 Oct 26;5(10):e13454. doi: 10.1371/journal.pone.0013454 (PMC2964313; doi:10.1371/journal.pone.0013454)
Supplement: Table S4 — Correlations between various SNPs and imputed classical alleles in the discovery dataset (0.01 MB PDF) [file pone.0013454.s004.pdf]

Table S4. Correlations between various SNPs and imputed classical alleles in the discovery dataset

	HLA-C*0501	HLA-B*4402	rs3129939G	DRB1*0301	DRB1*0401	DRB1*1303	DRB1*1501	rs9277535G
HLA-A*0201	0.22	0.20	-0.10	-0.14	0.18	-0.02	-0.06	0.007
HLA-C*0501		0.78	-0.07	-0.05	0.31	-0.02	-0.06	-0.02
HLA-B*4402			-0.06	-0.11	0.30	-0.03	-0.05	-0.02
rs3129939G				0.72	-0.16	-0.05	-0.22	-0.05
DRB1*0301					-0.16	-0.04	-0.18	-0.06
DRB1*0401						-0.05	-0.18	-0.04
DRB1*1303							-0.06	-0.006
DRB1*1501								-0.09
