# Supplementary material for: A Polymorphism in the HLA-DPB1 Gene Is Associated with Susceptibility to Multiple Sclerosis
Source: PLoS One. 2010 Oct 26;5(10):e13454. doi: 10.1371/journal.pone.0013454 (PMC2964313; doi:10.1371/journal.pone.0013454)
Supplement: Table S5 — A copy number variant at SNP rs2256583: numbers of heterozygous cases and controls with various C∶T allele ratios in the replication dataset (0.01 MB PDF) [file pone.0013454.s005.pdf]

Table S5. A copy number variant at SNP rs2256583: numbers of heterozygous cases and controls with various C:T allele ratios in the replication dataset

	C:T ratio		
	1:2	1:1	2:1
Cases	69	382	174
Controls	58	426	258
