# Supplementary material for: A Polymorphism in the HLA-DPB1 Gene Is Associated with Susceptibility to Multiple Sclerosis
Source: PLoS One. 2010 Oct 26;5(10):e13454. doi: 10.1371/journal.pone.0013454 (PMC2964313; doi:10.1371/journal.pone.0013454)
Supplement: Table S6 — Correlation between HLA-DPB1*0301 and rs9277535 in 422 MS cases (0.01 MB PDF) [file pone.0013454.s006.pdf]

Table S6. Correlation between HLA-DPB1*0301 and rs9277535 in 422 MS cases

	rs9277535 genotype		
	AA	AG	GG
DPB1*0301 positive	2	85	32
DPB1*0301 negative	194	100	9
