# Supplementary material for: A Polymorphism in the HLA-DPB1 Gene Is Associated with Susceptibility to Multiple Sclerosis
Source: PLoS One. 2010 Oct 26;5(10):e13454. doi: 10.1371/journal.pone.0013454 (PMC2964313; doi:10.1371/journal.pone.0013454)
Supplement: Figure S1 — A copy number variant at SNP rs2256583. A plot of area under the C- allele peak (y-axis) versus area under the T-allele peak (x-axis) for heterozygotes, showing three groups of individuals with C∶T ratios of 1∶2 (red), 1∶1 (green) and 2∶1 (blue). (0.16 MB PDF) [file pone.0013454.s007.pdf]

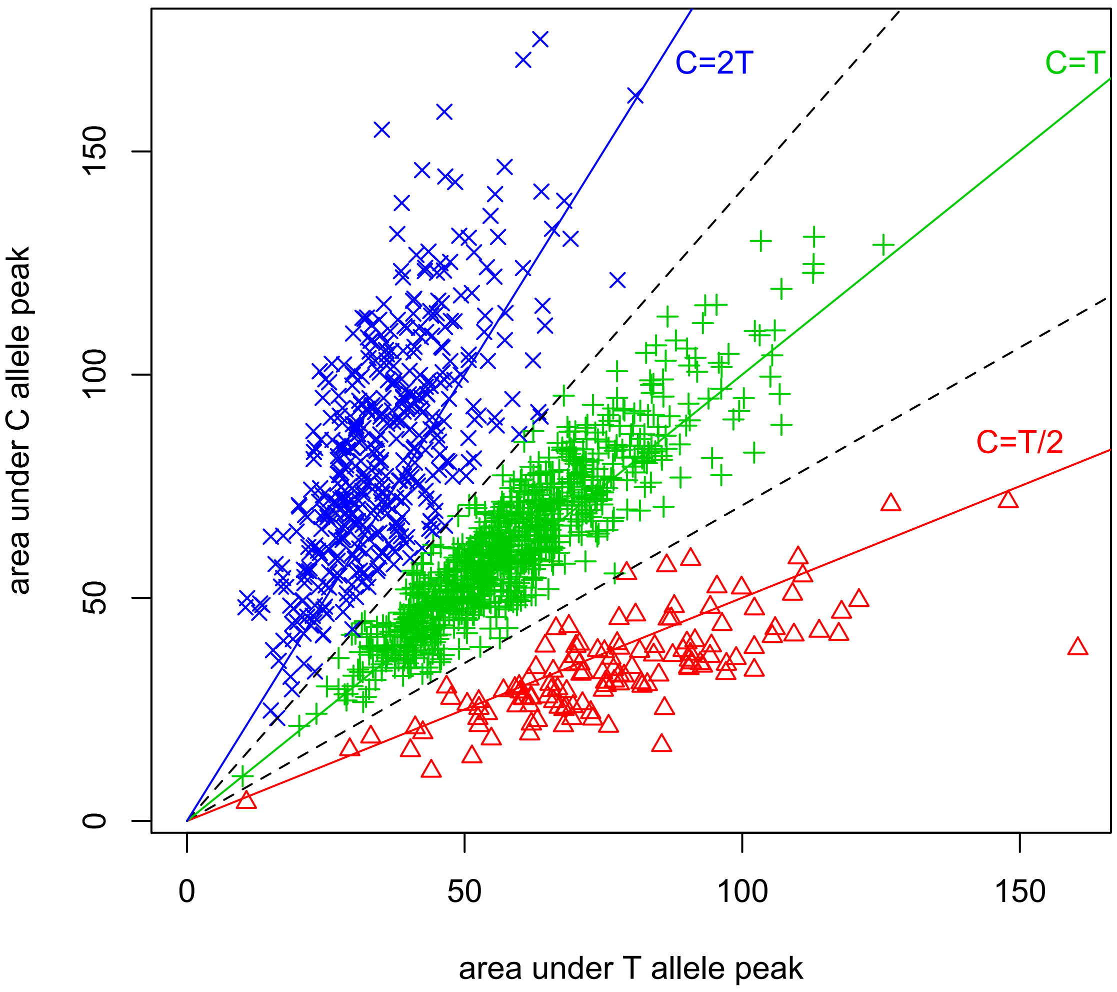

Figure S1. A copy number variant at SNP rs2256583 A plot of area under the C-allele peak (y-axis) versus area under the T-allele peak (x-axis) for heterozygotes, showing three groups of individuals with C:T ratios of 1:2 (red), 1:1 (green) and 2:1 (blue).
